# Supplementary material for: Measuring the burden of hundreds of BioBricks defines an evolutionary limit on constructability in synthetic biology
Source: Nat Commun. 2024 Jul 24;15:6242. doi: 10.1038/s41467-024-50639-9 (PMC11269670; doi:10.1038/s41467-024-50639-9)
Supplement: Supplementary file 1 — Supplementary Information [file 41467_2024_50639_MOESM1_ESM.pdf]

Supplementary Information

Measuring the burden of hundreds of genetic devices defines an evolutionary limit on constructability in synthetic biology

Noor Radde*, Genevieve A. Mortensen*, Diya Bhat, Shireen Shah, Joseph J. Clements, Sean P. Leonard, Matthew McGuffie, Dennis M. Mishler, Jeffrey E. Barrick#

Department of Molecular Biosciences, Center for Systems and Synthetic Biology, The University of Texas at Austin, Austin, TX 78712, U.S.A.

*These authors contributed equally to this work

#Correspondence: jbarrick@cm.utexas.edu

Contents

Supplementary Figures 1-8
Supplementary Table 1

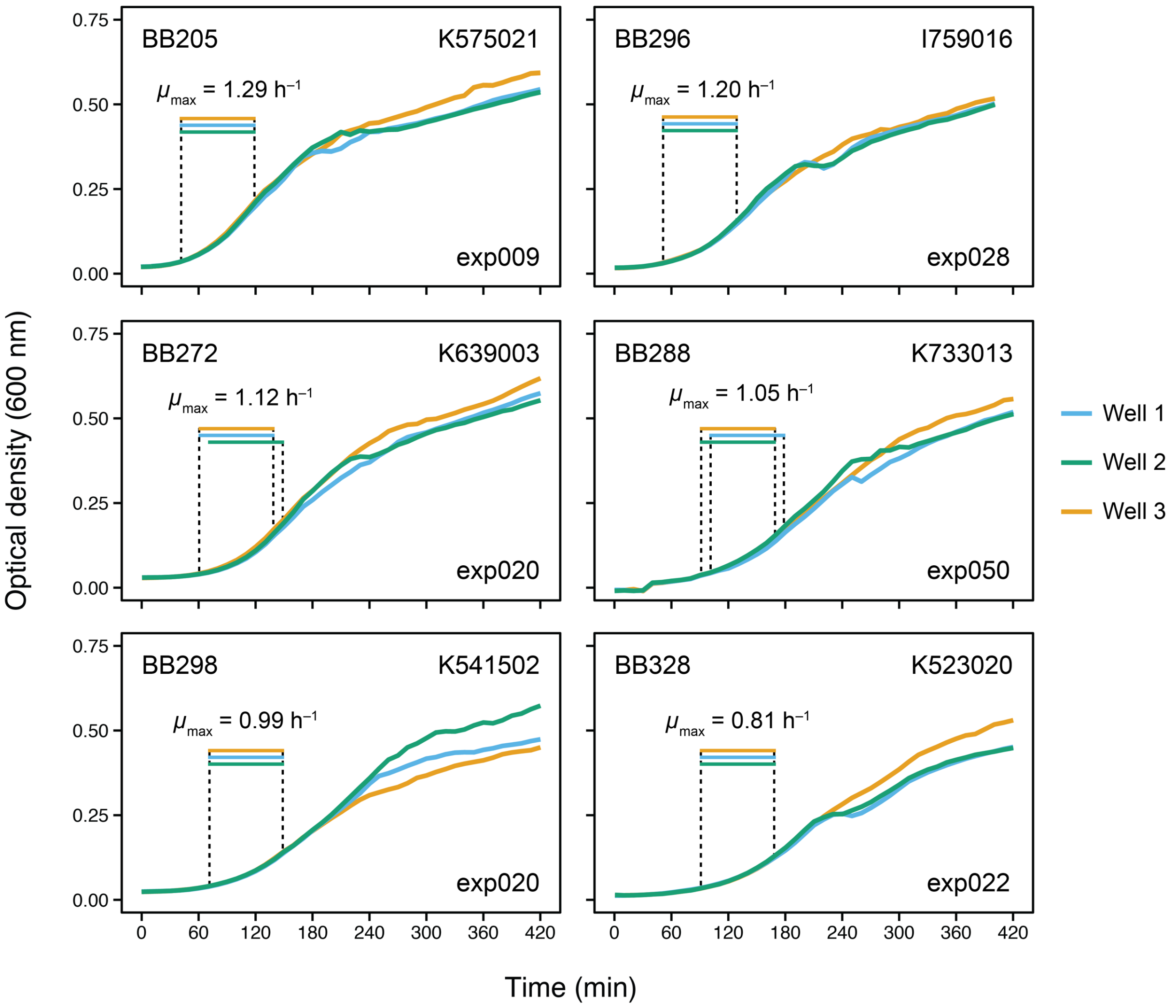

Supplementary Fig. 1. Representative growth curves used to measure BioBrick burden. Each panel shows data for a different *E. coli* strain (labeled in the upper left; ex: BB298) transformed with a plasmid containing a BioBrick part (labeled in the upper right; ex: K541502). For each strain, three growth curve replicates, each from a different well of the same microplate assay (labeled in lower right; ex: exp020) are shown. The BioBricks in all six of these strains were on the pSB1C3 backbone. Optical density values were measured every 10 minutes. They were corrected by subtracting the average value of the blank wells in the assay and then recentering the values for all three replicates to their average value across the replicates in the 0-60 min interval to account for well-to-well background variation (see **Methods**). Maximum specific growth rates (μ_{max}) for each well are written above three horizontal segments (colored the same way by well) indicating the 90-minute windows that gave the maximum fit growth rates. Source data are provided with this paper.

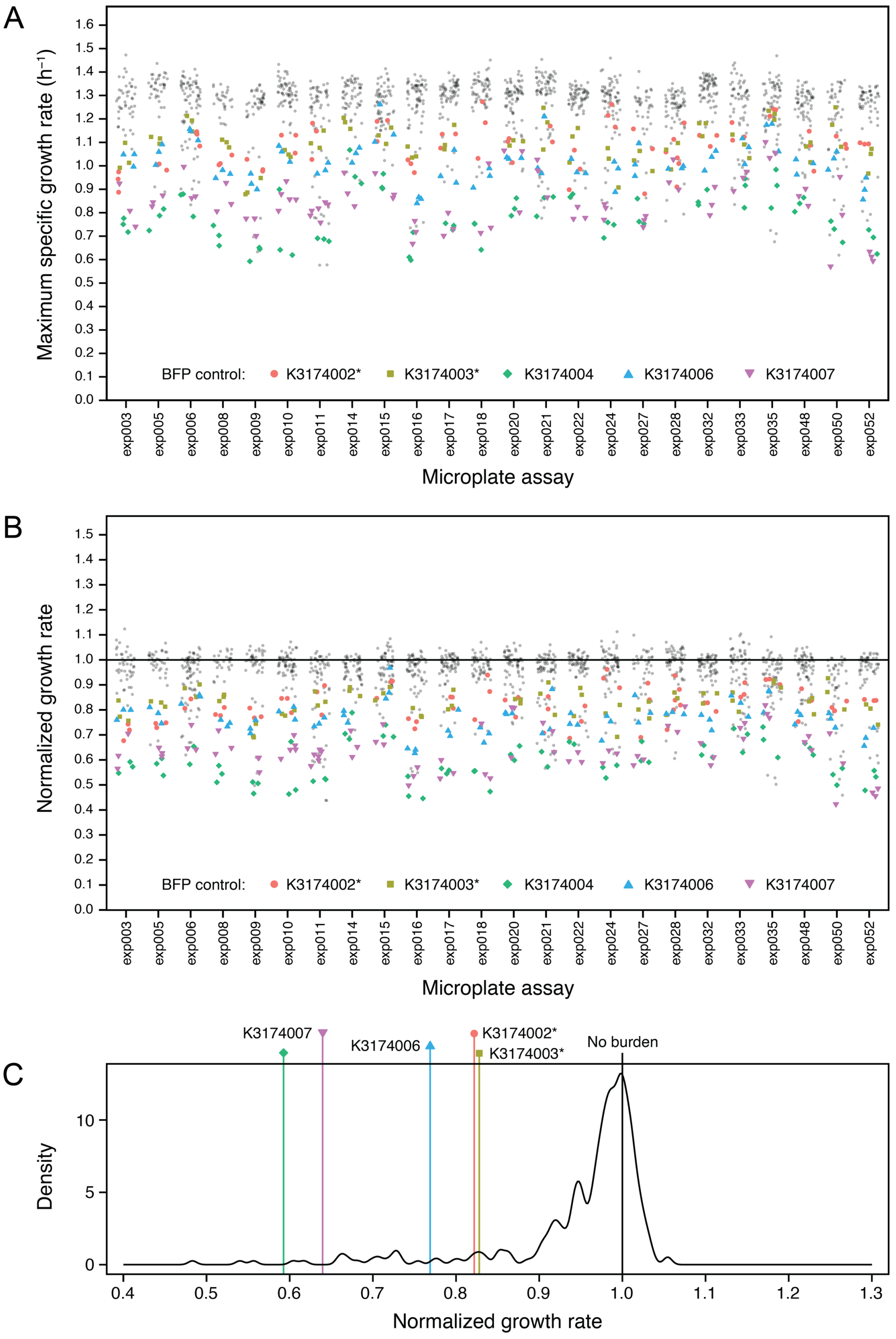

Supplementary Fig. 2. Growth rate measurements for all microplate assays. (**A**) Growth rates fit for each well containing an *E. coli* strain transformed with a BioBrick plasmid across 24 microplate assays. The five highlighted BioBricks are the BFP controls that were included in each assay. Cell stocks of the two starred BFP controls used in these assays had mutations that lowered their burden (**Supplementary Fig. 3**). (**B**) Normalized growth rates after correcting for variation between assays. (**C**) Final distribution of the mean normalized growth rates estimated for each BioBrick plasmid. The density is graphed using a Gaussian kernel with a bandwidth of 0.005. Source data are provided with this paper.

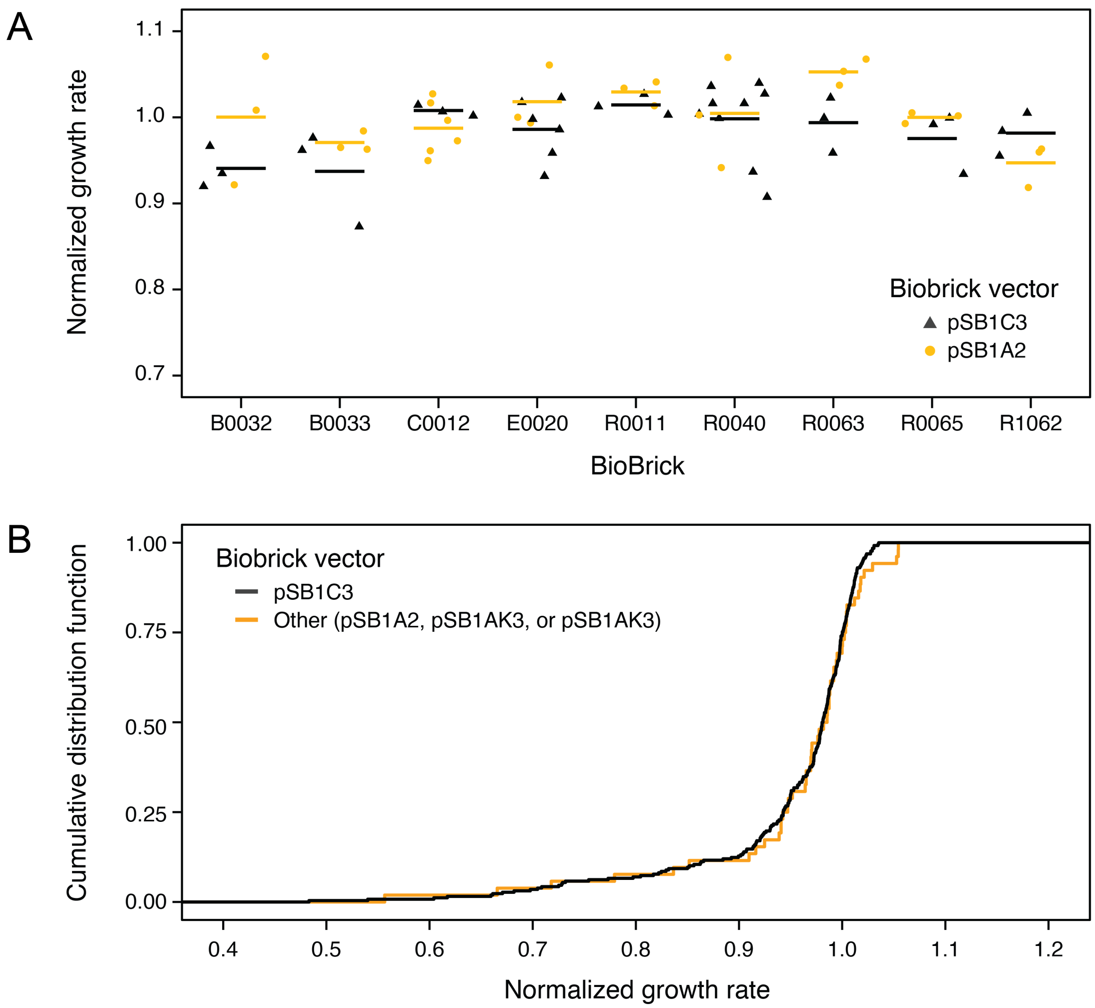

Supplementary Fig. 3. Comparison of growth rates measured for BioBricks in different plasmid backbones. (**A**) Growth rates fit for each well containing an *E. coli* strain transformed with one of the nine BioBricks that were tested in both the pSB1C3 and pSB1A2 backbones. Horizontal bars are mean values. (**B**) Cumulative distributions of the mean normalized growth rate values determined for the 259 BioBricks measured in the pSB1C3 backbone and the 52 measured in other backbones (40 in pSB1A2, 2 in pSB1AK3, and 1 in pSB3C5), excluding the BFP controls. Source data are provided with this paper.

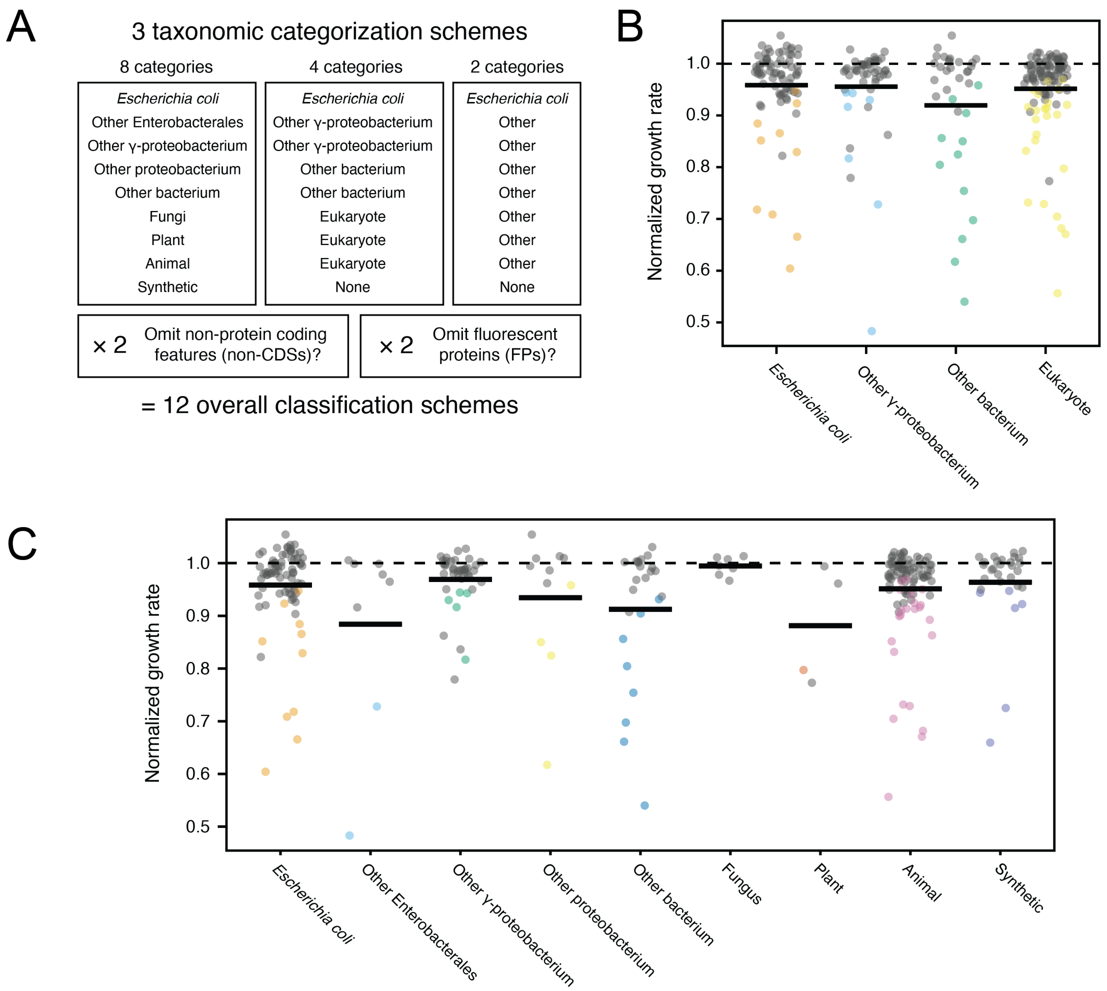

Supplementary Fig. 4. Correlation between BioBrick burden and type of organism of origin. (**A**) Three different schemes were used to categorize BioBricks based on the taxonomic classifications of the source organisms of their sequences (Methods). Names that align in rows show how the maximum of eight categories were collapsed to fewer categories in the other schemes. For each taxonomic categorization scheme, we tested variations that considered all combinations of including or omitting non-protein-coding features (non-CDSs) and including or omitting fluorescent proteins (FPs) in assigning each BioBrick an origin. BioBricks that were classified as having "None" as the source organism were omitted from each dataset that was analyzed. (**B**) Normalized growth rates of BioBricks classified according to the four-category taxonomic categorization scheme without omitting either non-CDSs or FPs. Points corresponding to the 59 burdensome BioBricks, for which the estimated burden was significantly greater than zero (one-tailed *t*-tests, Benjamini-Hochberg adjusted $p < 0.05$), are colored and others are gray. Horizontal bars show the average within each category. The dashed guideline is at a normalized growth rate of one (no burden). This scheme is the one case out of the twelve tested for which there was a significant difference in the chance that a part was classified as being burdensome with respect to the different organism categories (likelihood-ratio test comparing binomial models, $p = 0.047$) (Supplementary Table 1). (**C**) Normalized growth rates of BioBricks classified according to the eight-category taxonomic categorization scheme without omitting either non-CDSs or FPs. Symbols are as in B. Source data are provided with this paper.

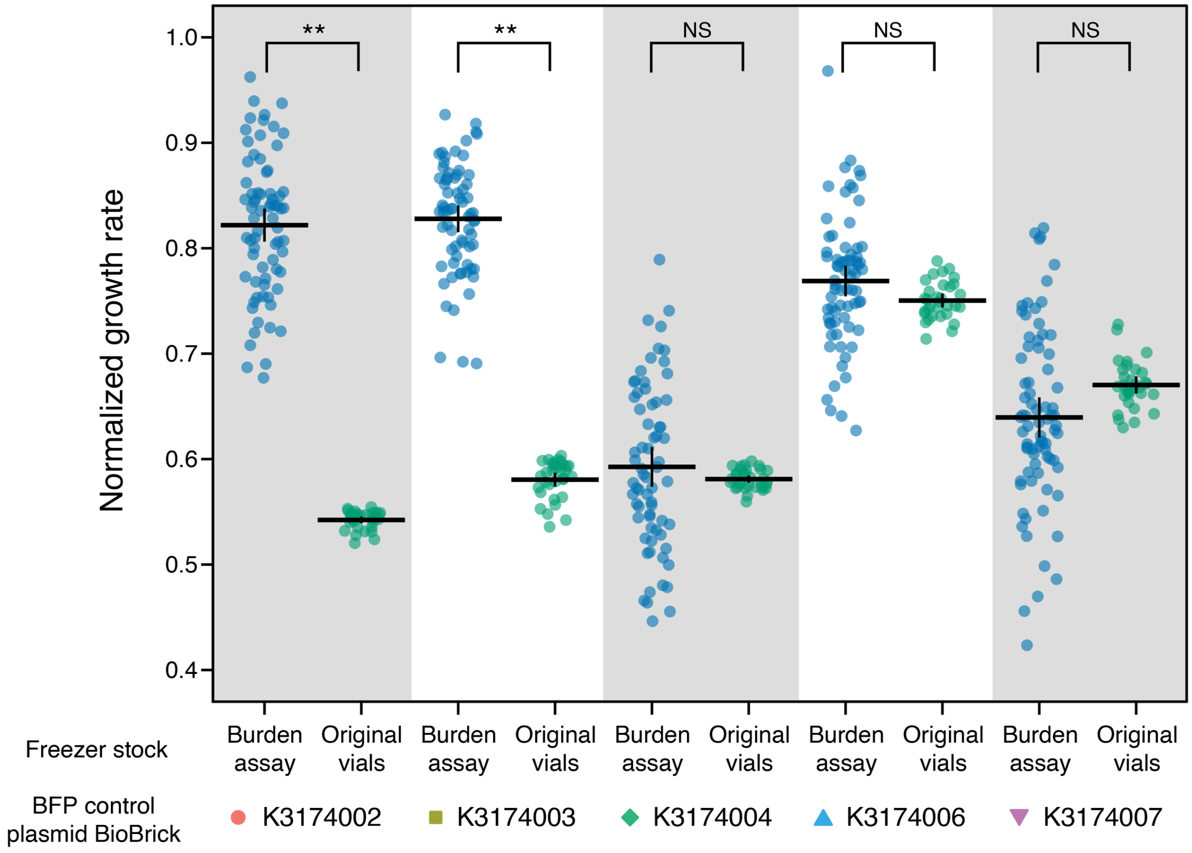

Supplementary Fig. 5. BFP plasmids in cell stocks used for microplate assays mutated to reduce burden. Blue points on the left in each set are normalized growth rate measurements for strains with BFP control strains containing the specified BioBrick plasmids from the freezer stocks used in all of the microplate burden assays (**Supplementary Fig. 2**, **Supplementary Fig. 7**). Green points on the right in each set are growth rate measurements of the BFP plasmid control strains grown from the original freezer vials of cells transformed with these plasmids, from which the stocks used in the burden assays were derived through picking colonies and regrowth. Measurements of the original freezer stock strains were normalized to the burden assay normalized growth rates using a linear regression that included only BFP control strains with BioBrick plasmids K3174004, K3174006, or K3174007. The stocks of cells with BioBrick plasmids K3174002 and K3174003 that were used in the burden assays were mutated in a way that greatly reduced their burden. Only the differences between freezer stocks for these two strains had normalized growth rates that were significantly different between the two sets (Bonferroni-corrected two-tailed *t*-tests; ** $p < 0.01$; NS, not significant, $p > 0.05$). Horizontal bars in each set of measurements show means and vertical bars show 95% confidence limits. Source data are provided with this paper.

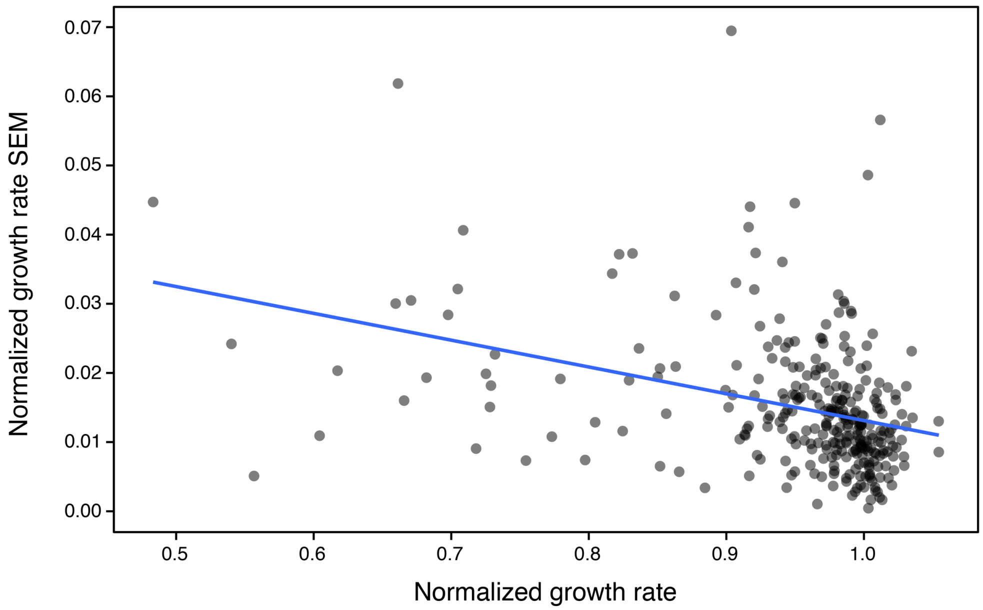

Supplementary Fig. 6. Growth rate measurements for BioBricks with higher burden exhibit more variability. The standard error of the mean (SEM) of the normalized growth rate measured for each of the 301 *E. coli* strains transformed with a different BioBrick plasmid is plotted versus its mean normalized growth rate. The blue trendline is the best-fit least-squares regression. Its slope is significantly different from zero (p = 2.0×10^{-11}, two-tailed t-test). Strains with lower growth rates (which carry plasmids with higher burden) exhibit more variation in their growth rates across replicate assays. Source data are provided with this paper.

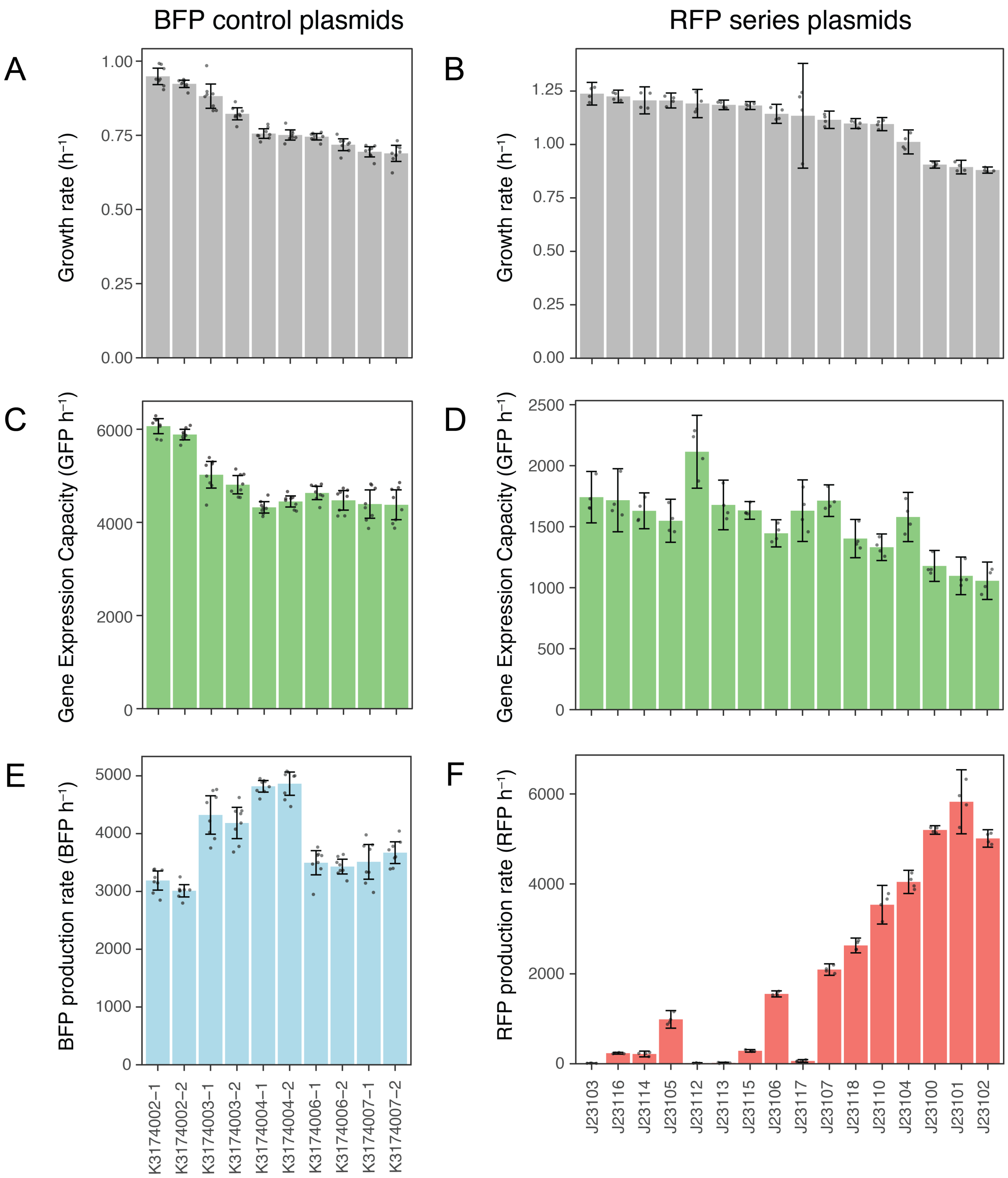

Supplementary Fig. 7. BFP and RFP plasmid measurements. (**A**, **B**) Growth rates of *E. coli* strains carrying either one of the BFP control plasmids with different promoter-RBS combinations or one of the RFP series plasmids with different promoters (**Fig. 6B**). (**C**, **D**) GFP production rates for the same strains. (**E, F**) BFP or RFP production rates for the same strains. In all panels, bars are means, error bars are 95% confidence intervals, and gray points are individual measurements. GFP, BFP, and RFP measurements are in arbitrary units and differed with the gain used in the instrument in each experiment. The values shown here were multiplicatively scaled in the main text figure (**Fig. 6C**). Source data are provided with this paper.

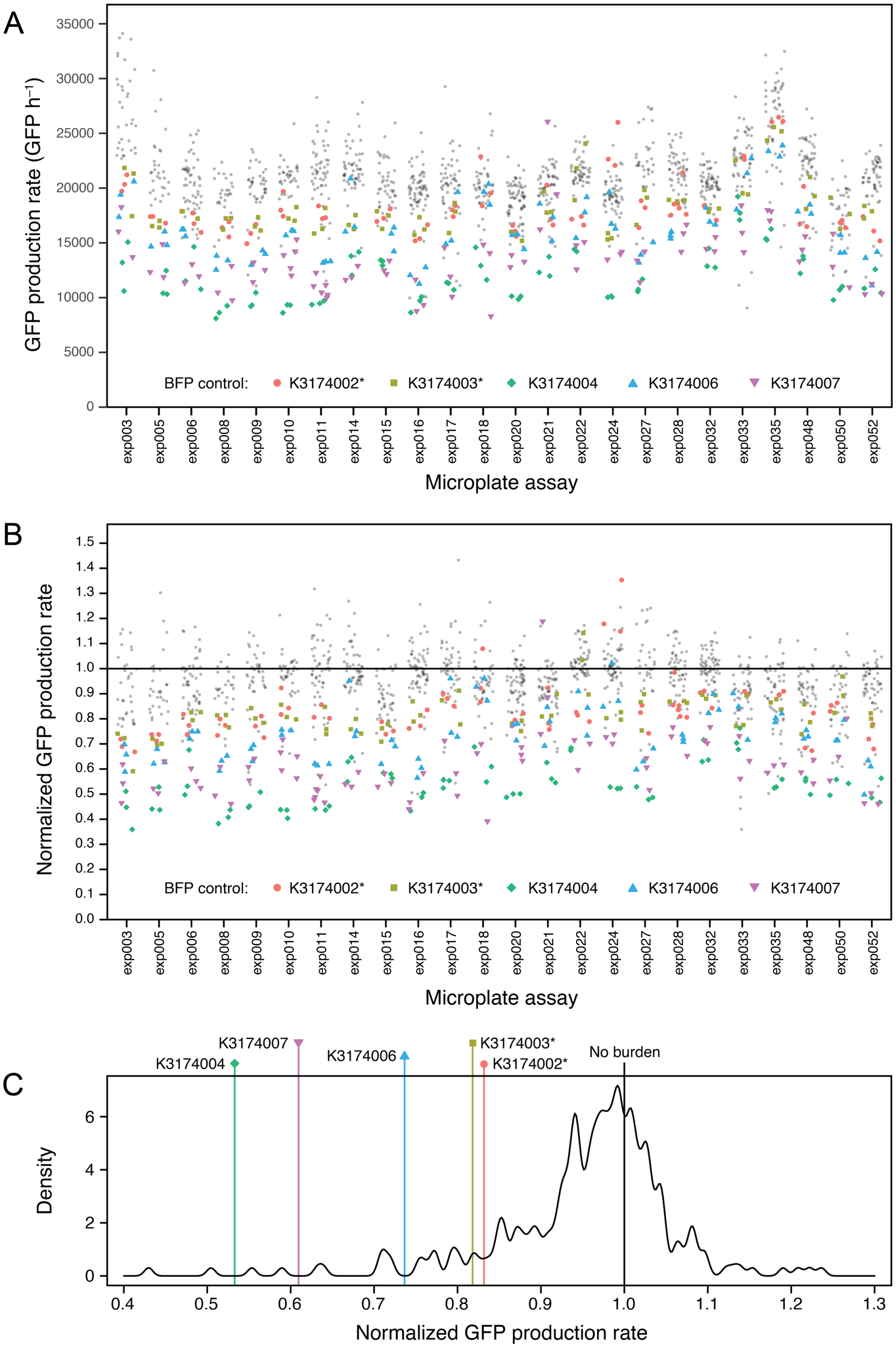

Supplementary Fig. 8. GFP production rate measurements for all microplate assays. (**A**) GFP production rates fit for each well containing an *E. coli* strain transformed with a BioBrick plasmid across 24 microplate assays. The five highlighted BioBricks are the BFP controls that were included in each assay. Cell stocks of the two starred BFP controls used in these assays had mutations that lowered their burden (**Supplementary Fig. 3**). (**B**) Normalized GFP production rates after correcting for variation between assays. (**C**) Final distribution of the mean normalized GFP production rates estimated for each BioBrick plasmid. The density is graphed using a Gaussian kernel with a bandwidth of 0.005. Source data are provided with this paper.

Supplementary Table 1. BioBrick organism of origin analysis

Categorization scheme*	Omit non-CDS*	Omit FPs*	BioBricks classified#	Statistical test	*p*-value	Sig&
8 categories	No	No	301	Kruskal-Wallis	0.210	
4 categories	No	No	271	Kruskal-Wallis	0.502	
2 categories	No	No	271	Kruskal-Wallis	0.301	
8 categories	Yes	No	221	Kruskal-Wallis	0.298	
4 categories	Yes	No	220	Kruskal-Wallis	0.603	
2 categories	Yes	No	220	Kruskal-Wallis	0.683	
8 categories	No	Yes	265	Kruskal-Wallis	0.250	
4 categories	No	Yes	234	Kruskal-Wallis	0.869	
2 categories	No	Yes	234	Kruskal-Wallis	0.774	
8 categories	Yes	Yes	165	Kruskal-Wallis	0.309	
4 categories	Yes	Yes	164	Kruskal-Wallis	0.588	
2 categories	Yes	Yes	164	Kruskal-Wallis	0.609	
8 categories	No	No	301 (59)	Binomial model LRT	0.132	
4 categories	No	No	271 (53)	Binomial model LRT	0.047	*
2 categories	No	No	271 (53)	Binomial model LRT	0.076	.
8 categories	Yes	No	221 (48)	Binomial model LRT	0.064	.
4 categories	Yes	No	220 (48)	Binomial model LRT	0.080	.
2 categories	Yes	No	220 (48)	Binomial model LRT	0.155	
8 categories	No	Yes	265 (53)	Binomial model LRT	0.364	
4 categories	No	Yes	234 (47)	Binomial model LRT	0.200	
2 categories	No	Yes	234 (47)	Binomial model LRT	0.720	
8 categories	Yes	Yes	165 (38)	Binomial model LRT	0.096	.
4 categories	Yes	Yes	164 (38)	Binomial model LRT	0.088	.
2 categories	Yes	Yes	164 (38)	Binomial model LRT	0.595	

*The first three columns specify one of the twelve schemes used for categorizing the source organisms of BioBricks based on the sequences of their genetic parts as described in Supplementary Fig. 4A. Abbreviations: CDS, protein-coding sequence; FP, fluorescent protein. #The number of the 301 total BioBricks tested that were analyzed in each scheme (*i.e.*, not including any BioBricks categorized as having "None" for the organism of origin). For each binomial model likelihood-ratio test (LRT), the number of analyzed BioBricks in the burdensome category that were included in that scheme is also shown in parentheses in this column. &Significance of the indicated statistical test. *P*-values ≤ 0.10 are marked with a dot, and *p*-values ≤ 0.05 are starred. Source data are provided with this paper.
