# Supplementary material for: Measuring the burden of hundreds of BioBricks defines an evolutionary limit on constructability in synthetic biology
Source: Nat Commun. 2024 Jul 24;15:6242. doi: 10.1038/s41467-024-50639-9 (PMC11269670; doi:10.1038/s41467-024-50639-9)
Supplement: Supplementary file 2 — Description of Additional Supplementary Files [file 41467_2024_50639_MOESM2_ESM.pdf]

Supplementary Data 1. Cell divisions during culture scale-up. Calculations used to estimate how many cell divisions occur during growth from a single engineered cell to a colony and then to cultures used at different laboratory and industrial scales.

Supplementary Data 2. BioBrick plasmid microplate assays. Growth rates and GFP production rates fit for every well of the microplate assays.

Supplementary Data 3. BioBrick plasmid statistics. Statistics compiled for each BioBrick from the results of all microplate assays and additional information about the BioBricks.

Supplementary Data 4. BioBrick plasmid sequencing results. Analysis of Illumina DNA sequencing results used to validate BioBrick plasmids.

Supplementary Data 5. BFP control and RFP series microplate assays. Growth rates, GFP production rates, and BFP/RFP production rates fit for every well of the microplate assays.

Supplementary Data 6. BFP control and RFP series statistics. Statistics compiled for each plasmid from the results of all microplate assays and additional information about the plasmids.
